# Genotype-refined 17OHP cut-offs diagnosing nonclassical CAH due to 21OH deficiency in children with premature pubarche

**DOI:** 10.1210/jendso/bvag162

**Published:** 2026-07-23

**Authors:** Nathalia Bordeira Chagas, Ayrton C Moreira, Margaret de Castro, Sonir R Antonini

**Affiliations:** Department of Pediatrics, Ribeirao Preto Medical School, University of Sao Paulo, Avenida Bandeirantes 3900, Ribeirao Preto 14049-900, Brazil; Department of Internal Medicine, Ribeirao Preto Medical School, University of Sao Paulo, Avenida Bandeirantes 3900, Ribeirao Preto 14049-900, Brazil; Department of Internal Medicine, Ribeirao Preto Medical School, University of Sao Paulo, Avenida Bandeirantes 3900, Ribeirao Preto 14049-900, Brazil; Department of Pediatrics, Ribeirao Preto Medical School, University of Sao Paulo, Avenida Bandeirantes 3900, Ribeirao Preto 14049-900, Brazil

**Keywords:** nonclassic congenital adrenal hyperplasia, 17-hydroxyprogesterone cut-offs, children, premature pubarche

## Abstract

**Context:**

Premature pubarche (PP) is a frequent reason for endocrine evaluation. Between 5% and 20% of children presenting with PP may have 21OHD nonclassic congenital adrenal hyperplasia (21OHD-NCAH).

**Objective:**

To define optimal basal and ACTH-stimulated 17OHP cutoffs for diagnosing 21OHD-NCAH in children with PP, using *CYP21A2* genotyping as the gold standard.

**Design, setting, and patients:**

A diagnostic accuracy study including 203 children with PP (median age: 7.5 years [6.4-8.3]; 85% female) who underwent ACTH stimulation testing. Biallelic pathogenic variants upon *CYP21A2* genotyping defined 21OHD-NCAH.

**Main outcome measure(s):**

Diagnostic performance of basal and post-ACTH 17OHP levels assessed by ROC curve analysis and thresholds defined according to the Youden index.

**Results:**

21OHD-NCAH was confirmed in 32 children (15.7%; 7.1 years [6.1-8.6]; 78% female). Basal 17OHP demonstrated excellent diagnostic accuracy (AUC = 0.98). A cutoff of 170 ng/dL (5.1 nmol/L) provided the best diagnostic balance, with 97% sensitivity and 91% specificity. A higher threshold of 410 ng/dL (12.4 nmol/L) achieved 100% specificity but reduced sensitivity (75%), whereas a lower cutoff of 118 ng/dL (3.5 nmol/L) yielded 100% sensitivity at the expense of specificity (84%). A post-ACTH 17OHP > 1104 ng/dL (33.4 nmol/L) yielded 100% sensitivity and specificity. Androgen levels overlapped substantially, and no clinical feature reliably distinguished 21OHD-NCAH from PP.

**Conclusion:**

Basal 17OHP provides excellent diagnostic accuracy for 21OHD-CAH in children with PP. A dual-threshold strategy is supported: 170 ng/dL (5.1 nmol/L) as optimal screening cutoff and 410 ng/d (12.4 nmol/L) as highly specific diagnostic threshold, obviating ACTH testing. These genotype-anchored, RIA-derived cutoffs are readily applicable in clinical settings using comparable assays.

Premature pubarche (PP), defined as the development of pubic or axillary hair before age 8 in girls and before age 9 in boys, is a common reason for referral to pediatric endocrinology (1). Most PP cases are due to (idiopathic) premature adrenarche. PP and premature adrenarche are not interchangeable terms. Premature adrenarche refers to the earlier-than-normal development of adrenarche in the presence of clinical signs of hyperandrogenism (greasy hair and skin, body odor, axillary hair) and elevated adrenal DHEAS levels (2). This diagnosis is confirmed after the exclusion of other severe forms of hyperandrogenism, including virilizing forms of congenital adrenal hyperplasia (CAH), adrenocortical tumors, and central precocious puberty (CPP) (in boys).

Among these, nonclassic congenital adrenal hyperplasia (NCAH) caused by 21-hydroxylase deficiency (21OHD-NCAH) is the most frequent condition, accounting for 5% to 20% of children evaluated for precocious pubarche (1, 3, 4).

21OHD-NCAH is an autosomal recessive disorder caused by biallelic pathogenic variants in the *CYP21A2* gene, resulting in milder 21-hydroxylase deficiency in adrenal steroidogenesis. This deficiency leads to the accumulation of 17-hydroxyprogesterone (17OHP) and increased adrenal androgen synthesis. Unlike patients with classical CAH, those with 21OHD-NCAH maintain sufficient aldosterone and cortisol synthesis to avoid adrenal crisis (5). The clinical phenotype is variable, but during childhood includes signs of mild androgen excess—such as PP, accelerated linear growth, advanced bone age, oily skin, and early-onset acne (6-8).

Early and accurate distinction between 21OHD-NCAH and premature adrenarche is essential for appropriate clinical management and assessment of long-term risk of hyperandrogenic manifestations. Although the measurement of 17OHP after the ACTH stimulation test is considered the diagnostic gold standard (5), its use may be limited by restricted availability in many countries. Consequently, basal 17OHP measurement is frequently employed as an initial screening tool. However, proposed basal cutoff values, typically ranging from 170 to 300 ng/dL (5.1-9 nmol/L), are extrapolated from adult or mixed-age cohorts (5, 9, 10).

To date, the diagnostic accuracy of basal and ACTH-stimulated 17OHP concentrations, using *CYP21A2* genotyping as the definitive reference, has been evaluated in only a limited number of pediatric studies. Establishing genotype-anchored biochemical thresholds represents a critical step toward improving diagnostic accuracy, reducing unnecessary ACTH testing, and informing practical algorithms for evaluation of PP. This study aimed to define optimal basal and post-ACTH 17OHP thresholds for diagnosing 21OHD-NCAH exclusively in children with PP, using *CYP21A2* genotyping as the diagnostic gold standard.

## Methods

### Ethical approval

The study was approved by the Institutional Ethics Committee of the Hospital das Clínicas, Ribeirao Preto Medical School, University of São Paulo (protocol no. 4.303.425). Written informed consent for genetic testing was obtained under amendment number 5.991.522.

### Study design and population

A retrospective diagnostic accuracy study involving 203 children evaluated for PP between January 1997 and December 2021 at a tertiary pediatric endocrinology center. PP was defined as pubic hair development before age 8 in girls and before age 9 in boys. We excluded patients with androgen-secreting tumors, non-21-hydroxylase forms of CAH, or other causes of hyperandrogenism.

**Figure 1:**
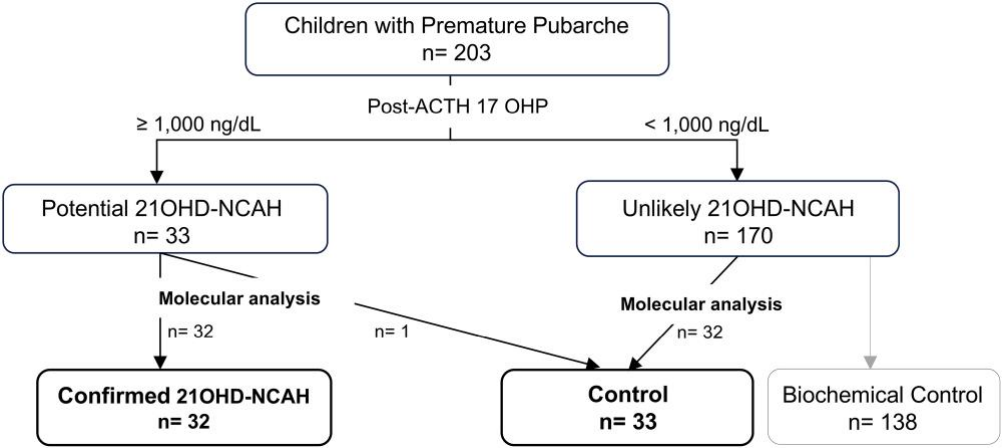
Flow diagram illustrating the study sample selection.

The study was structured in 2 phases (Fig. 1). In Phase 1, participants were stratified by a post-ACTH 17OHP cutoff of 1000 ng/dL (30 nmol/L), in accordance with Endocrine Society guidelines (5). *CYP21A2* genotyping was performed in all children with post-ACTH 17OHP greater than 1000 ng/dL (30 nmol/L) and in a subset of patients with post-ACTH 17OHP below this threshold. Molecular analysis was used as the gold standard for diagnosis. 21OHD-NCAH was defined as the presence of biallelic pathogenic variants (homozygous or compound heterozygous) that result in milder 21-hydroxylase deficiency. Controls carried either no *CYP21A2* variants or a single pathogenic variant in a heterozygous state. Optimal basal and stimulated 17OHP were defined exclusively in these genetically characterized groups.

In Phase 2, based on the optimal post-ACTH 17OHP cutoff identified in Phase 1, we redefined all 203 children with PP accordingly, and subsequent analyses focused on determining the most accurate basal 17OHP thresholds for diagnosing 21OHD-NCAH.

### Clinical and radiological assessment

Data were extracted from medical records. Anthropometric data were expressed as z-scores according to World Health Organization (WHO) growth standards for children aged 0-5 years (2006) and 5-19 years (2007). Growth velocity (cm/year) was calculated as the difference in height measurements obtained over a 1-year interval from the first clinical visit. Pubertal development was assessed according to Tanner staging.

Bone age was assessed via left-hand radiography using the Greulich–Pyle method. Skeletal maturation was expressed as delta bone age (bone age minus chronological age, in months).

### Hormonal assays

All patients underwent ACTH stimulation testing between 8:00 and 9:00 Am with 250 µg intravenous synthetic ACTH (Synacthen® Novartis, Basel, Switzerland). Blood samples for 17OHP were collected at baseline and 60 minutes post-ACTH. Serum 17OHP was measured by a specific radioimmunoassay (RIA) method, using previously ether-extracted and validated antibodies, as described previously (11, 12). The assay sensitivity was 15 ng/dL (0.45 nmol/L); intra- and interassay coefficients of variation were 3% to 4% and 7% to 16%, respectively. Plasma testosterone, androstenedione, and cortisol were determined by RIAs after extraction. The assay sensitivity and the intra- and interassay coefficient of variation were 1.2 mcg/dL (33.1 nmol/L), 5% and 10.5% for cortisol, 19 ng/dL (0.66 nmol/L), 5.6% and 17% for testosterone; and 4 ng/dL (0.14 nmol/L), 4% and 16% for androstenedione, respectively (13).

Cross-method agreement for 17OHP measurement between our specific RIA and liquid chromatography–tandem mass spectrometry (LC–MS/MS; Fleury Laboratory, São Paulo, Brazil) was assessed using a set of 30 routine clinical samples spanning a broad concentration range. Preanalytical conditions were identical for both methods. Passing–Bablok regression and the Cusum test were applied. The methods demonstrated good linear agreement (Cusum, *P* = .34), with a slope of 0.87 (95% CI, 0.61-1.04) and an intercept of −36.8 (95% CI, −61.3 to −2.8), indicating a slight constant bias without proportional deviation. The residual standard deviation was 1047.0, corresponding to limits of agreement of approximately ±2050 ng/dL, confirming overall analytical comparability between methods.

### Genotyping

Genomic DNA was extracted from peripheral blood leukocytes by QIAamp DNA blood kit (Qiagen, CA, United States). Molecular analysis was performed in sequential steps. A polymerase chain reaction (PCR) was used to amplify 2 large *CYP21A2* gene fragments, 2080 bp for the 5′UTR region and the other with 1420 bp for the 3′UTR region, which covers all transcribed exons. After confirming the amplification of these 2 fragments on agarose gel, a purification reaction of this material was performed to remove excess primers and unincorporated nucleotides, using the ExoSAP-IT™ PCR enzyme (ABI PRISM/PE Biosystems, Foster City, CA, United States). These 2 fragments were used to perform sequencing reactions using the Big Dye™ Terminator Cycle Sequencing Kits V3.1 Ready Reaction kit (ABI PRISM/PE Biosystems, Foster City, CA, United States). After the sequencing reaction, the samples were purified and precipitated using ethanol and isopropanol, dried in a dry bath, and resuspended in hydroformamide. They were then analyzed using an ABI3130 DNA sequencer (ABI PRISM/PE Biosystems). Sequence alignment was performed against the *CYP21A2* reference (Ensembl ENSG00000231852) using CodonCode Aligner software (CodonCode Corporation, Centerville, MA, United States).

Positive and negative DNA controls were included in all reactions (14). For patients diagnosed with 21OHD-NCAH, *CYP21A2* genotyping was additionally performed in their parents.

### Statistical analysis

Continuous variables were reported as medians and interquartile ranges; categorical variables as counts and percentages. Normality was tested using the Shapiro–Wilk test. Comparisons used the Mann–Whitney *U* test (continuous) and Fisher's exact test (categorical).

Receiver operating characteristic (ROC) curves were constructed to evaluate the diagnostic performance of basal and post-ACTH 17OHP. Sensitivity, specificity, area under the curve (AUC), and positive likelihood ratios (LR+) were calculated. The optimal cutoff value was determined using the Youden index, which maximizes the sum of sensitivity and specificity. All analyses were performed in R software (version 3.4.2), and a significance level of 5% was adopted.

## Results

A total of 203 children with PP were included in the analysis after excluding cases with adrenal or ovarian tumors, other forms of CAH, or exogenous testosterone exposure (Table 1). Among them, 32 patients (15.7%) were diagnosed with 21OHD-NCAH based on post-ACTH 17OHP concentrations >1000 ng/dL (30 nmol/L) and confirmed by *CYP21A2* genotyping. A comparison group consisted of 33 children with PP but without biallelic pathogenic variants (molecular control). Of these, only one child had post-ACTH 17OHP >1000 ng/dL (30 nmol/L). The remaining 138 patients were categorized in phase 2 as a second control group based on biochemical profile (biochemical control group) (Fig. 1). Of these 171 control patients with PP, 117 (68.4%) presented with DHEAS levels equal to or greater than 40 µg/dL (1 µmol/L) and were classified as having premature adrenarche. In the remaining 54 control patients with PP, DHEAS was below 40 µg/dL (1 µmol/L) and they were classified as having isolated PP. Of note, none of the 32 patients diagnosed with 21OHD-NCAH had undergone neonatal screening for 21OHD-CAH, as all were born before 2013, the year 21OHD-CAH was included in the universal public newborn program screening in Brazil. Therefore, neonatal 17OHP levels were unavailable among confirmed 21OHD-NCAH patients.

**Table 1:** Clinical demographic and hormonal profile for all 203 children, and comparison between 21OHD-NCAH and control groups

	(*n*)	All (203)	(*n*)	21OHD-NCAH (32)	(*n*)	Control (33)	*P* ^a^
General features							
Sex female (*n*, %)	172	85%	25	78%	30	91%	*.18*
Age at diagnosis (years)	203	7.5 [6.4-8.3]	32	7.1 [6.1-8.6]	33	7.8 [6.3-8.5]	.*77*
Pubarche age (years)	203	5 [5-6]	32	6 [5-6.8]	33	6.5 [5.5-7.3]	.*11*
Symptom duration (months)	203	12 [5-67]	32	12 [5.3-25]	33	10 [4-18]	.*39*
Height SD	194	1.2 [0.5-1.9]	32	1.5 [0.4-2.3]	31	1.0 [−0.2 to 1.8]	.*13*
BMI SD	194	1.8 [0.8-2.5]	32	2.0 [1.1-3.0]	31	1.0 [−0.5 to 2.1]	.***03***
Tanner B/G	203	1 [1-2]	32	1 [1-2]	33	1 [1-2]	.*52*
Tanner P	203	2 [2-3]	32	2 [2-3]	33	2 [2-3]	.*47*
Growth velocity (cm/year)	163	6.5 [5.5-7.6]	29	6.2 [5.4-7.1]	27	6.8 [5.4-7.5]	.*31*
Hormonal profile							
Basal							
Androstenedione (ng/dL) (nmol/L)	201	50 [33-75] *1.4 [1.15-2.6]*	32	100 [54-137] *3.5 [1.9-4.8]*	31	54 [28-88] *1.9 [1-3.1]*	***<***.***05***
DHEA-S (µg/dL) (µmol/L)	203	62 [30-99] *1.7 [0.8-2.7]*	32	81 [54-170] *2.2 [1.4-4.6]*	33	57 [23-77] *1.5 [0.6-2.1]*	***<***.***05***
Testosterone (ng/dL) (nmol/L)	201	32 [22-46] *1.1 [0.7-1.6]*	31	39 [28-57] *1.3 [1-2]*	33	34 [27-53] *1.2 [0.9-1.8]*	.*49*
ACTH test							
Basal 17 OHP (ng/dL) (nmol/L)	203	69 [41-153] *2.1 [1.2-4.6]*	32	740 [346-1371] *22.4 [10.5-39.8]*	33	82 [62-159] *2.5 [1.9-4.8]*	***<***.***05***
Post-ACTH 17 OHP (ng/dL) (nmol/L)	203	230 [167-470] *6.9 [5-14.2]*	32	4262 [2509-6255] *129 [76-189.3]*	33	360 [260-525] *10.9 [7.9-15.9]*	***<***.***05***
Cortisol (µg/dL) (nmol/L)	188	11 [8.4-15] *303 [232-414]*	28	10 [8.4-13] *276 [232-359]*	26	14 [7.4-17] *386 [204-469]*	.*32*
Post-ACTH cortisol (µg/dL) (nmol/L)	188	24 [19-30] *662 [524-828]*	28	17 [14-21] *469 [386-579]*	26	28 [21-32] *772 [579-883]*	***<***.***05***

Data are expressed as number of individuals, percentile, and median [interquartile range].

^*a*^Statistical comparisons were performed using the nonparametric Mann–Whitney *U* test for continuous data and Fisher's exact test for categorical data.

Laboratory evaluation revealed significantly higher median values of basal 17OHP, androstenedione, and DHEAS in 21OHD-NCAH patients than in controls (*P* < .05 for all comparisons). Post-ACTH 17OHP levels were markedly elevated in the 21OHD-NCAH group (4262 ng/dL [2509-6255] or 129.1 nmol/L [76.0-189.5]) compared to control (360 ng/dL [260-525] or 10.9 nmol/L [7.8-15.9], *P* < .05) (Table 1). Cortisol response to ACTH was lower in 21OHD-NCAH patients, with 35.7% showing suboptimal response (between 14-18 µg/dL or 385-495 nmol/L) and 25% showing inadequate peak cortisol (10-14 µg/dL or 275-385 nmol/L), compared with 4% of the control group for both categories (*P* < .05). All 21OHD-NCAH patients were treated with a low dose of glucocorticoid until the end of puberty to control the hyperandrogenism; however, none of the patients in our cohort required treatment of stress dosing. The management was individualized and based on clinical context rather than biochemical data alone.

To further explore genotype–phenotype correlations, patients were stratified into 3 genotype groups (A/C, B/C, and C/C) according to the degree of residual 21-hydroxylase activity, as previously described (15). We observed that patients with the C/C genotype (mild/mild variants) were more frequently represented among those with adequate adrenal reserve (post-ACTH cortisol >18 µg/dL or >495 nmol/L), whereas individuals with A/C and B/C genotypes (severe/mild and moderate/mild compound heterozygosity, respectively) were more prevalent in the group with lower cortisol responses (10-14 µg/dL or 275-385 nmol/L) (Table S1) (16).

Genetic analysis confirmed biallelic *CYP21A2* pathogenic variants in all 21OHD-NCAH cases, with the p.V281L variant being the most frequent (47% homozygous and 50% in compound heterozygous). In the control group, 72.8% had no detected variants and 27.2% carried a single-allele mutation, most commonly p.V281L (Table 2).

**Table 2:** Distribution of *CYP21A2* genotypes between 21OHD-NCAH and control groups

*CYP21A2* genotype	21OHD-NCAH (*n* = 32)
p.V281L/p.V281L	15 (47%)
p.V281L/p.30L	1 (3.1%)
p.V281L/p.P453S	1 (3.1%)
p.V281L/IVS2-13A/C > G	3 (9.4%)
p. V281L/p.R356W	3 (9.4%)
p.V281L/p.R124H	1 (3.1%)
p.V281L/p.R316X	1 (3.1%)
p.V281L/Cl6	1 (3.1%)
p.V281L/DEL8	1 (3.1%)
p.V281L/p.I172N	4 (12.5%)
p.P30L/p.I172N	1 (3.1%)

*CYP21A2* genotype	Control (*n* = 33)
WT/WT	24 (72.8%)
WT/p.V281L	7 (21.2%)
WT/IVS2.13A/C > G	1 (3%)
WT/p.Q318X	1 (3%)

Abbreviation: WT, wild type.

**Figure 2:**
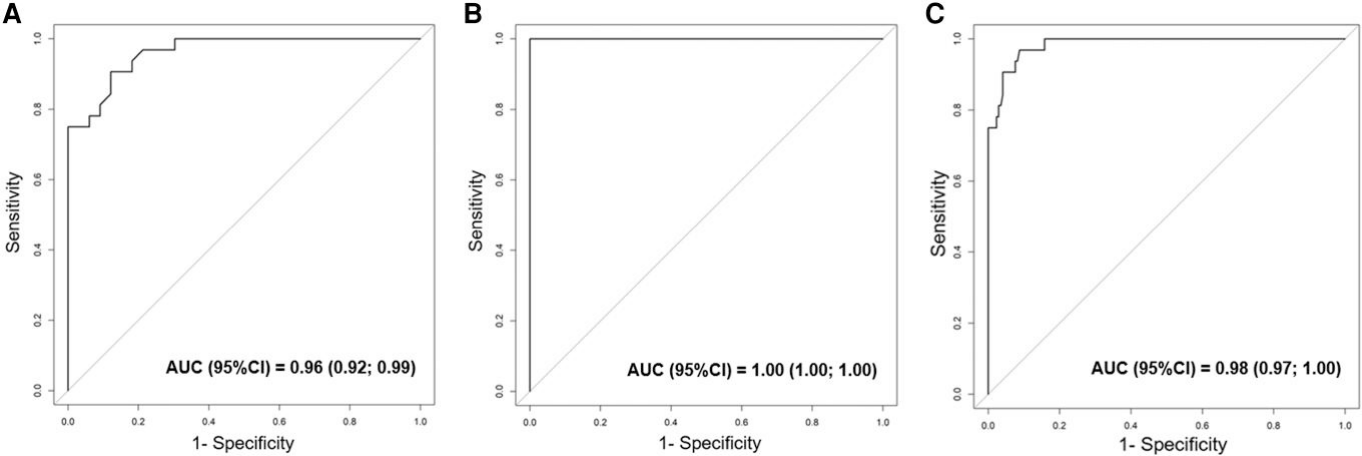
ROC curves demonstrating the diagnostic performance of (A) basal and (B) post-ACTH 17OHP levels for identifying 21OHD-NCAH patients among children with premature pubarche who underwent *CYP21A2* genotyping. (C) ROC curve demonstrating the diagnostic performance of basal 17OHP in all children with premature pubarche, with 21-OHDNCAH defined by post-ACTH 17OHP ≥ 1104 ng/dL (33.4 nmol/L).

ROC curve analysis using molecular diagnosis as the gold standard showed an AUC of 0.96 (95% CI 0.92-0.99) for basal 17 OHP and 1.00 for post-ACTH 17OHP (Fig. 2A and 2B, respectively). A basal 17OHP threshold of 117 ng/dL (3.5 nmol/L) provided 100% sensitivity but 70% specificity, whereas a threshold of 410 ng/dL (12.4 nmol/L) yielded 100% specificity and 75% sensitivity. A post-ACTH 17OHP of 1104 ng/dL (33.4 nmol/L) provided 100% sensitivity and specificity.

**Figure 3:**
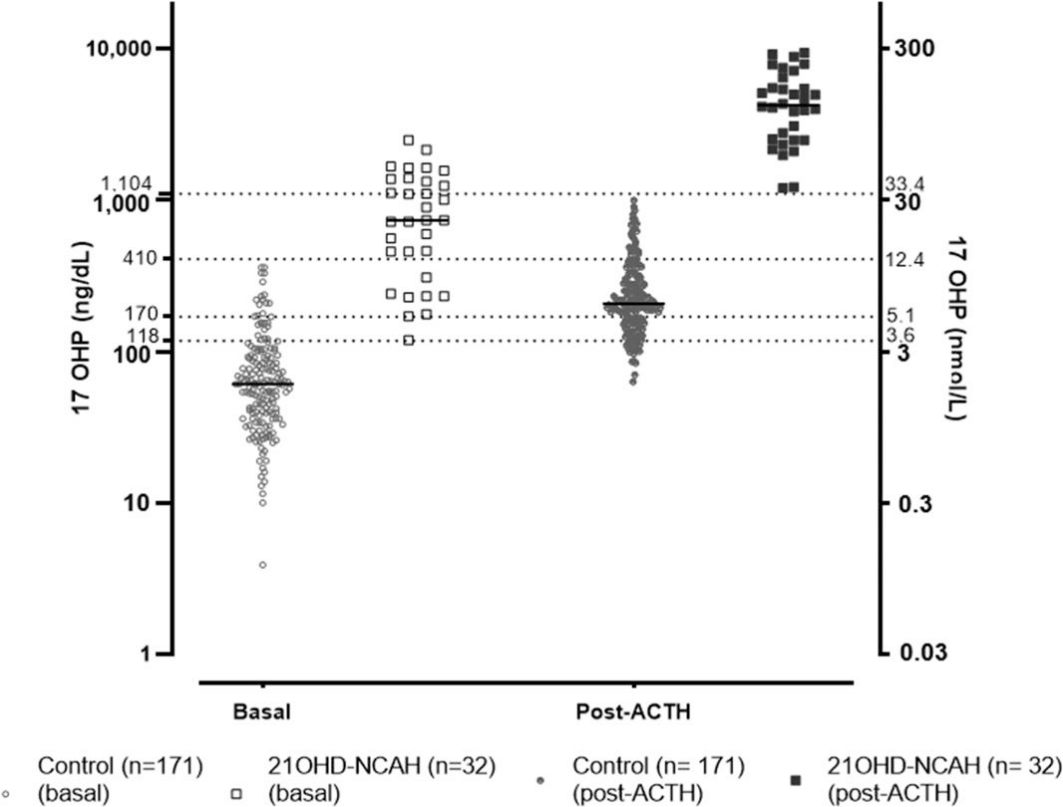
Individual basal and post-ACTH 17OHP concentrations in 21OHD-NCAH and control patients, with indicated cutoff values (118 ng/dL—3.6 nmol/L, 170 ng/dL—5.1 nmol/L, 410 ng/dL—12.4 nmol/L, and 1104 ng/dL—33.4 nmol/L).

In a subsequent phase that included all 203 patients, post-ACTH 17OHP >1104 ng/dL (33.4 nmol/L) was adopted as the diagnostic definition of 21OHD-NCAH. In this scenario, ROC analysis for basal 17OHP again demonstrated excellent diagnostic accuracy (AUC 0.98, 95% CI 0.97-1.00) (Fig. 2C). A basal cutoff of 118 ng/dL (3.6 nmol/L) showed 100% sensitivity and 84% specificity, while 410 ng/dL (12.4 nmol/L) maintained 100% specificity and 75% sensitivity. The optimal diagnostic balance, according to the Youden index, was achieved at a cutoff of 170 ng/dL (5.1 nmol/L) (sensitivity 97%, specificity 91%, and LR+= 11) (Table S2) (16). Adopting this baseline threshold, 16 out of 203 patients (7.9%) will still require ACTH testing to exclude 21OHD-NCAH. The distribution of individual basal and post-ACTH 17OHP concentrations in the 21OHD-NCAH and control groups is shown in Fig. 3. In Fig. S1 (16), we present a flowchart summarizing the diagnostic utility of basal and post-ACTH 17OHP levels to assess clinical progression in patients with PP over a 1-year follow-up in our cohort.

The 21OHD-NCAH and control groups were similar in sex distribution, with a predominance of females in both (78% vs 91%, respectively). The age at first evaluation was 7.1 years in the 21OHD-NCAH group and 7.8 years in the control group (*P* = .77). The age at pubarche onset was 6.0 years in 21OHD-NCAH patients and 6.5 years in controls (*P* = .11). Time from symptom onset to initial consultation did not differ significantly (12 months vs 10 months, respectively, *P* = .39). While no differences were observed in height *z*-scores (1.5 vs 1.0, *P* = .13), the frequency of tall stature was significantly higher in the 21OHD-NCAH group (34.5% vs 12%, *P* = .04). BMI *z*-score was significantly greater in 21OHD-NCAH patients than in controls (2.0 vs 1.0, *P* = .03), with a higher proportion of overweight or obese (78% vs 51.5%, *P* = .04) (Tables 1 and 3).

**Table 3:** Nutritional status and height classification based on BMI- and height-for-age *z*-scores according to WHO criteria in 21OHD-NCAH and control groups

Classification	21OHD-NCAH (*n* = 32)	Control (*n* = 33)	*P^b^*
Height *z*-score			
Short stature	1 (3%)	1 (3%)	*1*.*00*
Normal height	20 (62.5%)	26 (79%)	.*18*
Tall stature	11 (34.5%)	4 (12%)	.***04***
Not available	—	2 (6%)	*—*
BMI *z*-score			
Normal weight	7 (22%)	14 (42.5%)	.*11*
Overweight or obesity*^a^*	25 (78%)	17 (51.5%)	.***04***
Not available	—	2 (6%)	*—*

Data are presented as number of individuals (percentage).

^*a*^Overweight or obesity includes all categories above the normal BMI-for-age range (risk of overweight, overweight, obesity, and severe obesity) according to WHO BMI-for-age *z*-score classification (World Health Organization, 2006 and 2007).

^*b*^Statistical comparisons were performed using Fisher's exact test.

Clinical features such as axillary odor (68.8% vs 66.7%), axillary hair (40.6% vs 36.4%), and acne (40.7% vs 24.3%) were similarly distributed between the 21OHD-NCAH and control groups, respectively. Pubertal staging at diagnosis was comparable (Tanner stage 1 for breast/genital and stage 2 for pubic hair in both groups). During follow-up at the endocrinology unit, CPP was diagnosed in 34% of 21OHD-NCAH patients and 18% of controls (*P* = .17), and all were treated with GnRH analogs. Among 21OHD-NCAH patients who developed CPP, only 36.4% had initiated glucocorticoid therapy prior to CPP onset, whereas the remaining patients were diagnosed with CPP either before or shortly after the diagnosis of 21OHD-NCAH. Growth velocity during the first year after referral did not differ significantly between groups (6.2 vs 6.8 cm/year, *P* = .31), even when stratified by presence or absence of CPP. Similarly, delta bone age did not differ significantly between groups (27.0 vs 22.5 months; *P* = .15).

Comparisons between molecular and biochemical control subgroups showed no significant differences in most clinical and radiological features, except for BMI *z*-score, which was higher in the biochemical control subgroup (1.9 vs 1.0, *P* = .03) (Table S3) (16). However, BMI *z*-score did not differ between the biochemical control subgroup and 21OHD-NCAH patients (1.9 vs 2.0; *P* = .52). The frequency of tall stature remained highest in the 21OHD-NCAH group (34.5%) compared to both control subgroups (12% and 17.5%, respectively; *P* = .04) (Tables S3 and 4) (16).

Hormonal profiles were broadly comparable between control subgroups, with the exception of basal and post-ACTH 17OHP, which were higher in the molecular control subgroup (*P* < .05) (Table S3) (16). This finding likely reflects selection bias, as among individuals with post-ACTH 17OHP concentrations below 1000 ng/dL (30 nmol/L), those with higher values were preferentially selected for *CYP21A2* genotyping.

## Discussion

Accurate biochemical discrimination between children with PP and 21OHD-NCAH is essential for timely management. In the present study, both basal and ACTH-stimulated 17OHP concentrations demonstrated excellent diagnostic accuracy for identifying 21OHD-NCAH in children with PP. All genotype-confirmed 21OHD-NCAH cases exhibited post-ACTH 17OHP above 1104 ng/dL (33.4 nmol/L), reinforcing the diagnostic validity of the widely used 1000 ng/dL (30 nmol/L) threshold and supporting current Endocrine Society guidelines (5). *CYP21A2* analysis confirmed the predominance of the p.V281L variant, consistent with its well-established association with the nonclassic phenotype (15, 17).

Basal 17OHP also showed excellent diagnostic performance as an initial screening marker. A threshold of 170 ng/dL (5.1 nmol/L) provided optimal sensitivity (97%) and specificity (91%). Similar values have been reported for diagnosing 21OHD-NCAH in women with hyperandrogenism (9). Studies focused on pediatric cohorts (4, 18) suggested a threshold of 200 ng/dL (6 nmol/L); however, those cohorts exhibited a lower prevalence of 21OHD-NCAH. In our study, which had a higher prevalence of confirmed cases, 9% of children with 21-OHD (21OHD-NCAH) had basal 17OHP concentrations below this threshold. Ghizzoni et al (19) identified 282 ng/dL (8.5 nmol/L) as the optimal cutoff (sensitivity 84%, specificity 95%) in children with PP. Applying this threshold to our population would have resulted in a missed diagnosis in 22% of genetically confirmed 21OHD-NCAH patients. These findings highlight the need for population-specific cutoffs and demonstrate that thresholds derived from mixed-age or adult cohorts may not be appropriate for the pediatric population.

A basal 17OHP cutoff of 410 ng/dL (12.4 nmol/L) demonstrated 100% specificity, suggesting its utility as a diagnostic discriminator between 21OHD-NCAH and PP when clinical suspicion is high. Maffazioli et al (20) identified 540 ng/dL (16.3 nmol/L) as an effective diagnostic threshold for distinguishing 21OHD-NCAH from polycystic ovary syndrome in adult women. These findings collectively indicate that optimal cutoffs in pediatric populations tend to be lower than those reported in adults.

In settings where ACTH stimulation testing may not be feasible, a basal 17OHP threshold of 118 ng/dL (3.6 nmol/L) may be adopted as a screening tool given its 100% sensitivity. However, its lower specificity would increase the rate of false-positive results, highlighting the inherent balance between broad case detection and the risk of unnecessary referrals. Diagnostic thresholds should thus be adapted to the local clinical context, balancing access, accuracy, and resource availability.

Regarding the methodology for 17OHP measurement, although LC-MS/MS provides increased analytical specificity and precision, its availability remains limited in many clinical laboratories. RIA remains widely used and has demonstrated satisfactory diagnostic accuracy for 21OHD-NCAH, particularly when method-specific thresholds are applied (4, 18-20). Our comparative analysis between RIA and LC-MS/MS showed good linear agreement, with only a slight constant bias and no significant proportional deviation, further supporting the applicability of RIA-derived thresholds in centers where LC-MS/MS is not routinely available.

Beyond 17OHP, we also analyzed the potential diagnostic contribution of adrenal androgens. Although androstenedione has been identified as a useful marker for 21OHD-NCAH in prior studies (4, 21, 22), and DHEAS typically shows limited diagnostic utility (21-23), our findings revealed significantly higher median levels of both markers in 21OHD-NCAH patients. However, substantial overlap with controls limited their discriminatory value. These findings reaffirm the central role of 17OHP for diagnosis and suggest that androstenedione and DHEAS, despite their elevation, may offer limited incremental diagnostic accuracy.

In individuals with 21OHD-NCAH, the post-ACTH cortisol peak may be suboptimal, typically ranging from 14 to 18 µg/dL (385-495 nmol/L) (24). In our cohort, the 21OHD-NCAH group exhibited lower post-ACTH cortisol peaks compared with controls, with 35.7% showing suboptimal responses. Similar findings have been reported in 28% to 60% of patients with 21OHD-NCAH in previous studies (19, 20, 24, 25).

In our cohort, subsets of patients in both 21OHD-NCAH and control groups were subsequently diagnosed with GnRH-dependent precocious puberty. However, median 17OHP and androgen levels were comparable between those with and without CPP. Accordingly, these subgroups were not analyzed separately. In clinical settings, differentiating PP as the earliest sign of puberty from its presentation as a manifestation of 21OHD-NCAH may be challenging, particularly in children with obesity or borderline biochemical values. For this reason, some authors recommend evaluating children with clinical features of hyperandrogenism or early pubertal onset to exclude 21OHD-NCAH (26).

The prevalence of 21OHD-NCAH in our PP cohort (15.7%) is consistent with prior pediatric reports (0-40%) and likely reflects underlying population allele frequencies (27). No single clinical feature reliably distinguished 21OHD-NCAH from idiopathic PP, although tall stature was more common among 21OHD-NCAH patients than among controls. High frequency of overweight and obesity (>70%) was observed in both groups, in line with global epidemiologic trends. Despite recognized associations between adiposity and early adrenarche or bone maturation (28), bone age advancement did not differ significantly between groups, thereby offering limited diagnostic utility.

Strengths of our study include the consistent use of a single assay method throughout the study period, minimizing analytical variability; molecular confirmation of all 21OHD-NCAH cases; and genotyping of selected controls to ensure accurate classification, particularly in children with PP and elevated post-ACTH 17-OHP levels. As in other tertiary-center cohorts, the prevalence of 21OHD-NCAH in our study may not fully reflect that of the general population.

The retrospective design of our study may have limited some analyses. Additionally, using the RIA rather than the LC–MS method to measure 17OHP could limit the generalizability of these results. However, the thresholds established in this study, derived from RIA data and anchored to *CYP21A2* genotyping, can be applied confidently in clinical settings using comparable methodologies, as demonstrated by the strong linear agreement between RIA and LC–MS/MS. Due to the retrospective design of our study and the fact that all patients with confirmed 21OHD-NCAH were born before 2013, when 21OHD-CAH was included in the universal public newborn screening program in Brazil, neonatal 17OHP levels were unavailable for these patients. It has been reported that some patients with 21OHD-NCAH already present with mildly to moderately elevated 17OHP levels at birth, which persisted over the following months after birth. This finding could be an early diagnostic window, avoiding delayed diagnosis (29-31).

## Conclusion

**Figure 4:**
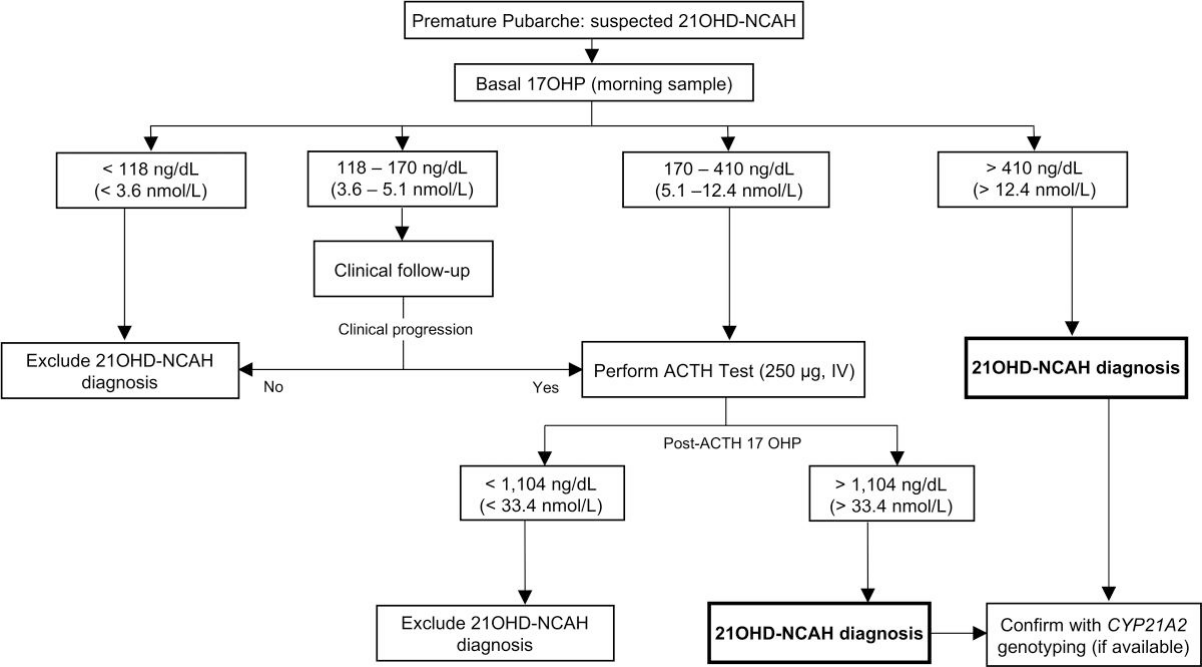
Proposed diagnostic algorithm for the evaluation of children with premature pubarche and suspected 21OHD-NCAH based on basal and post-ACTH 17-hydroxyprogesterone (17OHP) levels found in the present study.

This study provides 17OHP concentrations that optimize diagnostic accuracy for 21OHD-NCAH. Overall, our results support a dual-threshold strategy for basal 17OHP in children with PP: using 170 ng/dL (5.1 nmol/L) as an optimal screening cutoff, and 410 ng/dL (12.4 nmol/L) as a highly specific diagnostic threshold that may obviate the need for ACTH stimulation (Fig. 4). Thus, in pediatric populations, when ACTH testing is unavailable, basal 17OHP remains a robust and practical screening tool, especially when interpreted within genotype-informed frameworks.

## Data Availability

The data that support the findings of this study are available on request from the corresponding author.
